# Simultaneous Isolation of Circulating Nucleic Acids and EV-Associated Protein Biomarkers From Unprocessed Plasma Using an AC Electrokinetics-Based Platform

**DOI:** 10.3389/fbioe.2020.581157

**Published:** 2020-11-05

**Authors:** Juan Pablo Hinestrosa, David J. Searson, Jean M. Lewis, Alfred Kinana, Orlando Perrera, Irina Dobrovolskaia, Kevin Tran, Robert Turner, Heath I. Balcer, Iryna Clark, David Bodkin, Dave S. B. Hoon, Rajaram Krishnan

**Affiliations:** ^1^Biological Dynamics, Inc., San Diego, CA, United States; ^2^Departments of Translational Molecular Medicine and Sequence Center, John Wayne Cancer Institute, Santa Monica, CA, United States; ^3^Cancer Center Oncology Medical Group, La Mesa, CA, United States

**Keywords:** biomarkers, cell-free DNA, extracellular vesicles, plasma, personalized medicine, multi-omics, liquid biopsy, AC electrokinetics

## Abstract

The power of personalized medicine is based on a deep understanding of cellular and molecular processes underlying disease pathogenesis. Accurately characterizing and analyzing connections between these processes is dependent on our ability to access multiple classes of biomarkers (DNA, RNA, and proteins)—ideally, in a minimally processed state. Here, we characterize a biomarker isolation platform that enables simultaneous isolation and on-chip detection of cell-free DNA (cfDNA), extracellular vesicle RNA (EV-RNA), and EV-associated proteins in unprocessed biological fluids using AC Electrokinetics (ACE). Human biofluid samples were flowed over the ACE microelectrode array (ACE chip) on the Verita platform while an electrical signal was applied, inducing a field that reversibly captured biomarkers onto the microelectrode array. Isolated cfDNA, EV-RNA, and EV-associated proteins were visualized directly on the chip using DNA and RNA specific dyes or antigen-specific, directly conjugated antibodies (CD63, TSG101, PD-L1, GPC-1), respectively. Isolated material was also eluted off the chip and analyzed downstream by multiple methods, including PCR, RT-PCR, next-generation sequencing (NGS), capillary electrophoresis, and nanoparticle size characterization. The detection workflow confirmed the capture of cfDNA, EV-RNA, and EV-associated proteins from human biofluids on the ACE chip. Tumor specific variants and the mRNAs of housekeeping gene *PGK1* were detected in cfDNA and RNA isolated directly from chips in PCR, NGS, and RT-PCR assays, demonstrating that high-quality material can be isolated from donor samples using the isolation workflow. Detection of the luminal membrane protein TSG101 with antibodies depended on membrane permeabilization, consistent with the presence of vesicles on the chip. Protein, morphological, and size characterization revealed that these vesicles had the characteristics of EVs. The results demonstrated that unprocessed cfDNA, EV-RNA, and EV-associated proteins can be isolated and simultaneously fluorescently analyzed on the ACE chip. The compatibility with established downstream technologies may also allow the use of the platform as a sample preparation method for workflows that could benefit from access to unprocessed exosomal, genomic, and proteomic biomarkers.

## Introduction

The goal of personalized medicine is to individualize care pathways, tailor screening, treatment, disease monitoring, and prevention recommendations to one’s genomic and proteomic makeup (Crowley et al., 2013; Diaz Jr., and Bardelli, 2014; Lewis et al., 2015; Cohen et al., 2017; De Rubis et al., 2019). Scientific discoveries that drive our understanding of an individual’s cellular and molecular processes rely on a combination of insights based on genomic and proteomic technologies. These often require biomarker-specific purification workflows (DNA, RNA, and proteins) that are performed on multiple isolation platforms, use highly pre-processed biological material, and lengthy analytical procedures that limit routine clinical use (Gold et al., 2015; Volik et al., 2016; Cohen et al., 2018; Aggarwal et al., 2019).

Today, comprehensive profiling of a single blood sample could require the use of three distinct technologies, each using a separate isolation method to analyze one single class of biomarkers: circulating cell-free tumor DNA (ctDNA), tumor RNA, and proteins (Lo Cicero et al., 2015; Möhrmann et al., 2018). With the incorporation of new classes of biomarkers, such as extracellular vesicles (EVs) and their dedicated isolation workflows, analytical toolsets continue to grow in size and complexity, hampering translation to routine testing (Théry et al., 2018). Thus, an important step toward accessible personalized medicine could be the development of a workflow capable of simultaneous isolation and analysis of DNA, RNA, and EV-associated protein biomarkers in minimally processed samples (Vaidyanathan et al., 2018).

**FIGURE 1:**
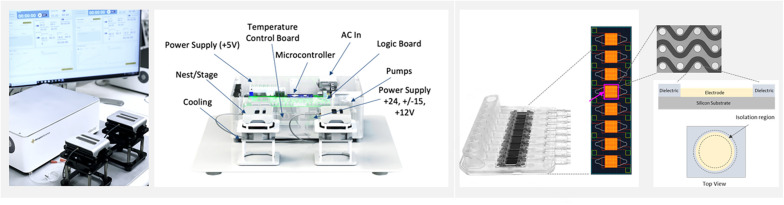
The ExoVerita system consists of an instrument and a consumable cartridge that encases a microfluidic chip. The instrument **(left)** controls and monitors AC Electrokinetics (ACE) run parameters. The chip **(right)** is split into eight arrays, with each array housing approximately 1,000 circular platinum electrodes that transmit capture signal. For on-chip detection experiments, one array is used per each sample; for isolation experiments, to maximize capture yield, all eight arrays are used per each sample.

The work presented here describes the characterization of a novel isolation platform, Verita (Biological Dynamics, CA), to perform rapid and simultaneous isolation of the three different types of biomarkers directly from blood-based matrices using an AC Electrokinetics (ACE) powered microelectrode array (ACE chip). The ACE field essentially functions as a band-pass filter; the field’s properties could be altered to enable enrichment of isolated biomaterial for biomarkers in a specific size range (Sonnenberg et al., 2013; Ibsen et al., 2015). During the isolation phase, the ACE field is generated on the chip using a specific voltage/frequency algorithm and microelectrode array design (Figure 1 and Supplementary Material). The field creates dipole on affected particles and directs them to specific locations on a microelectrode array (Oh et al., 2009; Krishnan et al., 2011; Ibsen et al., 2017; Turner et al., 2018).

The above principle is also applied for the workflows that analyze biological material that is enriched for cell-free DNA (cfDNA) and EVs bearing DNA, RNA, and proteins in unprocessed and minimally processed samples. Following the capture, biomarkers of interest are characterized either on the chip using fluorescent detection or eluted off the chip for secondary analysis with technologies such as PCR and NGS. On-chip detection of cancer biomarkers using the ACE method, as well as in-situ and downstream analysis of the captured biomarkers, have been demonstrated by multiple research groups (Sonnenberg et al., 2014; Manouchehri et al., 2016; Ibsen et al., 2017; Lewis et al., 2018, 2019, 2020). The objective of this work was to evaluate the feasibility of using a single-chip, multi-omic workflow from the perspective of applications in personalized medicine.

## Materials and Methods

### Ethics Statement

The prospective collection of human blood samples from cancer donors and healthy controls was approved through an Institutional Review Board and performed under Western IRB approved protocols. The samples and donor information were handled according to the Declaration of Helsinki. Written, informed consent for research use was obtained from individuals at participating institutions.

### Alternating Current Electrokinetic Microelectrode Array Chip (ACE Chip)

**FIGURE 2:**
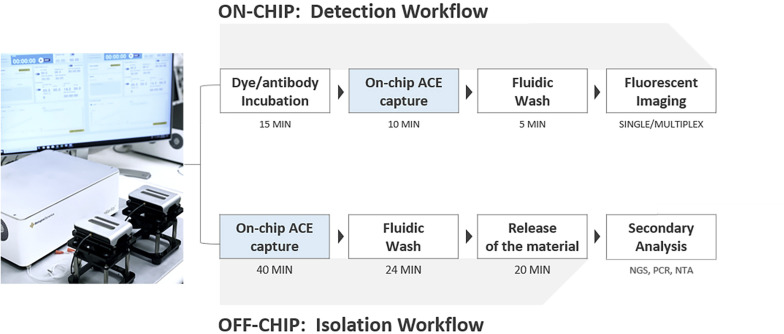
Verita Platform detection and isolation workflows. To detect biomarkers, antibodies, or dyes are incubated with samples, followed by ACE capture, washing, and imaging. Additional washes and antibody incubations can be performed for indirect immunofluorescence (**Detection workflow, top**). To isolate biomarkers, ACE capture is performed, followed by washing with an elution buffer, biomarker release from the electrodes, and collection of released materials (**Isolation workflow, bottom**).

The ACE chip used on the Verita platform is a microelectromechanical systems (MEMS) fabricated device with dimensions of 14 mm × 52 mm organized into an 8-array configuration, with each array consisting of ∼1,000 platinum circular electrodes coated with a hydrogel. For isolation workflow, minor changes in the chamber design were used to merge eight individual array chambers (used for detection workflow) into one single chamber. A basic schematic of ACE chip construction and a description of the principles of AC electrokinetic isolation of particles are shown in Supplementary Figure S1. The ACE chip is optimized to work with physiological solutions (conductivities 10–15 mS/cm). A schematic of the Verita platform with the detection and isolation workflows is shown in Figure 2.

### Reagents

NoLimits DNA fragments, YOYO-1 Iodide, and SYTO RNASelect Green Fluorescent Cell Stain were purchased from Thermo Fisher Scientific (Waltham, MA, United States). Human genomic DNA (gDNA) was purchased from Promega Corporation (Madison, WI, United States). Endotoxin-free Dulbecco’s phosphate buffered saline (PBS), molecular grade water, and saponin detergent were obtained from EMD Millipore (Burlington, MA, United States). Tris-EDTA, nuclease-free water was obtained from VWR (Radnor, PA, United States). UltraCruz Blocking Agent and antibodies directed against CD63 (clone MX-49.129.5), TSG101 (clone c-2), and glypican-1 (GPC-1; clone 4D1) were obtained from Santa Cruz Biotechnology (Dallas, TX, United States). Directly conjugated anti-programmed death ligand-1 antibody (PD-L1; clone 2746) was obtained from Biotium (Fremont, CA, United States). Fluorophore conjugated, affinity-purified F(ab)_2_ goat anti-mouse IgG was purchased from Jackson ImmunoResearch Laboratories (West Grove, PA, United States). K_2_EDTA plasma used for spiked gDNA and EV experiments was purchased from Innovative Research (Novi, MI, United States). Quantitative multiplex reference standard gDNA (HD701) was purchased from Horizon Discovery (Cambridge, United Kingdom). Cerebrospinal fluid was obtained from Gemini Bio-Products (West Sacramento, CA, United States).

### Plasma Preparation

Whole blood from cancer donors was prospectively collected in K_2_EDTA (Vacutainer Plastic Plus EDTA Blood Collection Tubes, Becton Dickinson, Franklin Lakes, NJ, United States) tubes and processed to plasma within 4 h of collection. The blood was processed into plasma by centrifuging blood tubes at 300 × g for 10 min at 23°C, followed by the transfer of the supernatant to a new conical tube and centrifuging again at 4,300 × g for 20 min at 23°C. Retrospectively collected cancer plasma specimens were also procured from BioIVT (Westbury, NY, United States) and Discovery Life Sciences (Los Osos, CA, United States), and stored at -80°C. Retrospective samples were thawed and spun at 4,300 g for 20 min at 23°C to remove any remaining cell debris or aggregated proteins immediately prior isolation on the ACE chip. Clinical characteristics of cancer donors are provided in Supplementary Table S1.

### Fluorescence Microscopy

Images were captured using a monochrome CMOS camera (FLIR, Wilsonville, OR, United States), and a BX41 microscope equipped with a Uplan FLX 4X objective (Olympus, Tokyo, Japan. Illumination was provided via a mercury vapor short arc lamp (Excelitas, Covina, CA, United States), and filtered using appropriate fluorescence bandpass filters and dichroic beamsplitters (Semrock, Rochester, NY, United States). All images were acquired with a 2,000 ms exposure at two different gains, one at zero and another at 24. For display purposes, images were loaded into Image J2 software (Rueden et al., 2017) and normalized to the same scale using the set display range feature.

### Co-detection of EV-Associated Proteins and Nucleic Acids

For simultaneous detection of cfDNA and exosomal proteins plasma samples prepared as described above were diluted 1:1 with 0.5 × PBS (undiluted samples were used for cfDNA-only detection experiments in Supplementary Figure S2). Either nucleic acid dyes (YOYO-1 or SYTO RNASelect) or primary antibodies directed against biomarker proteins of interest, together with a fluorophore-conjugated secondary antibody, were added directly to the diluted plasma samples and incubated for 15 min at 37°C [CD63, 1:400; PD-L1, 1:200; GPC-1, 1:1,000; Alexa Fluor 488-F(ab’)_2_ Goat anti-Mouse IgG, 1:750]. Samples were loaded onto the ACE chip and a signal of 7 Vpp and 14 kHz was applied to the chip for 10 min. Following capture, unbound material was washed off the chip with TE buffer for an additional 5 min at 10 μL/min with the same signal parameters. Chips were analyzed for the presence of each of the EV-associated proteins and cfDNA via fluorescence microscopy followed by image display and normalization using ImageJ2.

Luminal protein TSG101 visualization experiments used the following modified protocol. Unlabeled plasma samples were loaded onto the ACE chip, and a signal of 10 Vpp and 14 kHz was applied for 14 min. Following a TE buffer wash of 10 min at 3 μL/min, the ACE signal was turned off, and 0.1% saponin diluted into 1X PBS was flowed through the chip at 3 μL/min for 10 min. Antibody against TSG101 was diluted into UltraCruz Blocking Reagent and flowed through the ACE chip at 3 μL/min for 10 min. The fluid flow was then stopped, and the chip incubated for an additional 60 min at room temperature. To remove unbound antibodies, the ACE chip was washed with 1X PBS at 3 μL/min for 10 min. Fluorophore-conjugated secondary antibody was then flowed onto the chip and incubated for an additional 60 min, washed with 1X PBS, then analyzed as described above.

### Capture and Isolation of Cell-Free DNA Using the ACE Chip

A plasma sample was loaded into the ACE chip at a rate of 10 μL/min for 4 min to fill the flow cell. Once the flow cell was filled, the sample flowed through the chip at 3 μL/min for 40 min while an ACE signal of 8.5 Vpp and 14 kHz was applied for a total of 120μL processed on the chip. With the ACE signal still active, the flow cell was washed with Wash Solution 1 (Biological Dynamics) for 12 min at 3 μL/min followed by Elution Buffer 2 (Biological Dynamics) for 12 min at 3 μL/min. The electrical signal was turned off, and the chip was incubated at room temperature for 20 min to allow the cfDNA to detach from the electrodes. Approximately 35 μL of purified sample was removed from the flow cell.

### Capture and Isolation of EVs Using the ACE Chip

ACE chips were initially prepared, and plasma samples were loaded as described above. After the flow cell was filled, an electrical signal of 7 Vpp, 14 kHz was applied for 40 min while flowing plasma through the cell at 3 μL/min. The flow cell was washed with Elution Buffer 1 (Biological Dynamics) for 30 min at 3 μL/min. The electrical signal was turned off, and the chip incubated at room temperature for 20 min to allow the EVs to detach from the electrodes. Approximately 35 μL of purified sample was then removed from the flow cell.

### Polymerase Chain Reaction

PCR and RT-PCR were performed on isolated samples using the ViiA7 Real-Time PCR System (Thermo Fisher Scientific). DNA amplification was performed in a 20 μL reaction containing 10 μL of the Luna Universal qPCR Master Mix (New England BioLabs, Ipswich, MA, United States), 4 μL of nuclease-free water, 5 μL of DNA template or isolated sample directly following isolation, and 1 μL of primer/probe mix for mutation detection. The PCR reaction conditions were as follows: initial denaturation for 1 min at 95°C, then 45 cycles of denaturation (95°C for 15 s), and extension (60°C for 30 s). Sequences of the primers and probes for the *ACTB* gene were the following: Forward: 5’-AAG ACA GTG TTG TGG GTG TAG-3’; Reverse: 5’-AGACCTACTGTGCACCTACT-3’; Probe: 5’-VIC-TGTAAAGCGGCCTTGGAGTGTGTA-MGBNFQ-3’. Primers and probe for KRAS G12D mutation detection were purchased from Thermo Fisher Scientific (KRAS_521_mu, Assay ID: Hs000000051_rm, Cat. # A44177).

RNA amplification using RT-PCR was a one-step process performed in a 20 μL reaction containing 10 μL of the one-step reaction mix from the Luna Universal Probe One-Step RT-qPCR Kit (New England BioLabs, United States), 1 μL of Luna Warm Start RT Enzyme mix, 1 μL of nuclease free water, 7 μL of RNA template or isolated sample and 1 μL of the primer/probe mix for *PGK1* (Assay ID: Hs99999906_m1, Cat. # 4351370, Thermo Fisher Scientific). The amplification reaction conditions were as follows: reverse transcription for 10 min at 55°C, followed by initial denaturation for 1 min at 95°C, then 45 cycles of denaturation (95°C for 15 s) and extension (60°C for 30 s). Total RNA Control (Human, Cat. #4307281, Thermo Fisher Scientific) was used as a positive control.

### Next-Generation Sequencing

NGS was performed on isolated samples using an Illumina MiSeq sequencer (Illumina, San Diego, CA, United States) and the AmpliSeq for Illumina Cancer HotSpot Panel v2 for target amplification of regions that are commonly mutated in human cancer genes as well as the AmpliSeq Library PLUS and AmpliSeq UD Indexes for library preparation and index ligation. The library was prepared using 12 μL of sample, according to manufacturer’s protocols. Sequencing was performed in a 2 × 151 bp read fashion using the MiSeq Reagent Kit v2 (300-cycles) with 5% PhiX as control. FASTQ files were analyzed using the DNA Amplicon app version 2.1.1 (Illumina) with options selected for BWA aligner, RefSeq annotation, indel realignment, and variant caller depth filter for 100×.

### EV Isolation From Cell Culture Media

EVs were isolated from the cell culture supernatants of the cell lines from ATCC (Manassas, VA), MDA-MB-231 (ATCC HTB-26), and AsPC-1 (ATCC CRL-1682) by centrifugation at 3,000 RCF for 20 min followed by centrifugation at 100,000 RCF for 2 h. The EVs were then resuspended in 1X PBS and stored at -80°C, and hereafter are referred to as cell culture EVs.

### Particle Size Characterization

EV samples were analyzed with tunable resistive pulse sensing (qNano, Izon Science Ltd, Christchurch, NZ) using a NP150 nanopore membrane at a 47 mm stretch. The concentration of particles was standardized using multi-pressure calibration with 110 nm carboxylated polystyrene beads at a concentration of 1.2 × 10^13^ particles/mL.

### Electrophoretic Analysis of DNA and Proteins

DNA base-pair distribution and protein contamination were analyzed on a 2100 Bioanalyzer (Agilent Technologies, Santa Clara, CA, United States) using High Sensitivity DNA and Protein 230 kits, respectively, according to the manufacturer’s instructions.

### Scanning Electron Microscopy

Following sample capture, the ACE chip was washed with molecular grade water and frozen at -80°C for 48 h. Immediately before imaging, the chip was coated with 1 nm iridium using an Emitech K575x Sputter Coater (Quorum Technologies, Kent, United Kingdom). Scanning electron microscope images were acquired using a FEI Quant FEG 250 (Thermo Fisher Scientific). Acquisition settings are shown on the bottom banner of each image presented.

### Silica Column-Based Isolation of Cell-Free DNA

DNA from the AsPC-1 cell culture EVs and one cancer donor sample was isolated using the QIAamp Circulating Nucleic Acid Kit (Qiagen N.V., Hilden, Germany), further referred to as QNA, according to the manufacturer’s instructions from 120 μL sample input and isolated into 50 μL of buffer AVE.

## Results

### Simultaneous Capture and Detection of DNA, RNA, and EV-Associated Protein Biomarkers From Lung Cancer Donor Plasma With the ACE Chip

**FIGURE 3:**
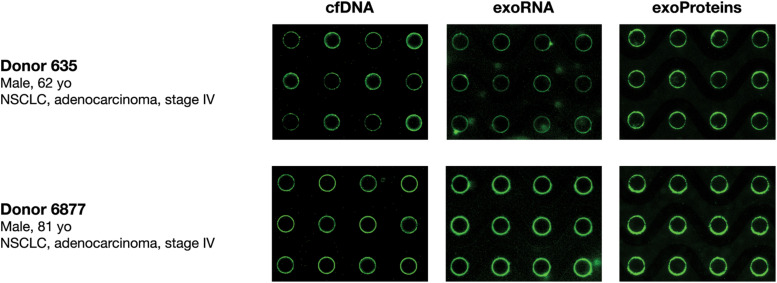
Simultaneous isolation and detection of nucleic acids (cfDNA, exoRNA) and EV-associated protein biomarkers from lung cancer donor plasma. Fluorescence imaging of chips to detect cfDNA (YOYO-1; **left panels**), exosomal RNA (SYTO RNAselect; **middle panels**), and exosome-associated proteins (anti-CD63 antibodies; **right panels**) following isolation from two NSCLC K_2_EDTA donor plasma samples (Donor 635 top, Donor 6877 bottom) onto the ACE chip.

The capability of the Verita platform to simultaneously isolate cfDNA, exosomal RNA (exoRNA), and exosomal protein in plasma was evaluated using fluorescent imaging. Biomarker capture and on-chip detection of the biomarkers was performed in plasma samples collected from two donors diagnosed with Non-Small Cell Lung Cancer (NSCLC). Fluorescent dyes and antibody specific for CD63 revealed a pattern consistent with the capture of cfDNA, exoRNA, and EV-associated proteins over the circular electrodes as shown in Figure 3.

The capability of the Verita platform to capture DNA from diverse specimen types (blood, serum, plasma, and cerebrospinal fluid) was investigated by spiking 50 pg/μL gDNA into each solution and running the detection workflow. Fluorescent signals detected on the ACE chip indicated the presence of DNA following capture from all four solutions tested (see Supplementary Figure S2). Previously published data showed that the fluorescent intensity is proportional to the input DNA concentration (Krishnan et al., 2011; Turner et al., 2018) and demonstrates the utility of our platform for physiologically relevant cfDNA concentrations in cancer patient plasma (Wu et al., 2002; Volik et al., 2016).

#### Characteristics of DNA Isolated by the ACE Chip on the Verita Platform

**FIGURE 4:**
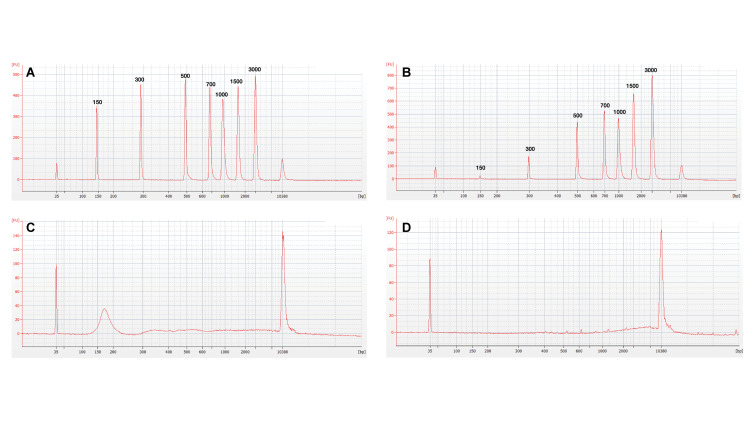
ACE chips can preferentially isolate long cell-free DNA fragments. Capillary electrophoresis profiles for analysis of DNA size in eluates before and after ACE chip capture. **(A)** Profile of 1× TE spiked with NoLimits DNA. **(B)** Profile of K_2_EDTA plasma spiked with NoLimits DNA after ACE chip capture and isolation. Endogenous DNA profiles using a lung cancer donor K_2_EDTA plasma sample (Donor 9488) **(C)** Before isolation, **(D)** After isolation.

To examine the size selectivity of the cfDNA capture, several size fractions of NoLimits DNA were spiked into K_2_EDTA plasma and captured on and isolated from the chip. Capillary electrophoresis was performed to determine the size distribution of DNA both before capture and following isolation. Results revealed that DNA >500 bp exhibited similar relative peaks both before (Figure 4A) and after ACE capture and isolation (Figure 4B). However, the 300 bp pre-capture peak was reduced in size, and the 150 bp fragments were largely absent from the isolated DNA. This result shows that the size selectivity of the chip can be biased toward capture of cfDNA longer than 300 bp.

To verify that spiked NoLimits DNA accurately reflected the behavior of endogenous cfDNA, patient donor plasma was isolated using the QNA kit and compared to direct isolation for the ACE chip. The isolated DNA from the QNA kit (Figure 4C) exhibited both high and low molecular weight peaks with the lower molecular weight peak representing the 160–180 bp DNA fragments commonly associated with apoptotic DNA (Sunami et al., 2008; Marzese et al., 2013; Zonta et al., 2015). Capillary electrophoresis analysis of the cfDNA following isolation using the chip demonstrated selection for large base pair fragments similar to the NoLimits DNA (Figure 4D). These results indicate that the ACE chip selectively isolates long cell-free DNA (>300 bp) from plasma samples.

**FIGURE 5:**
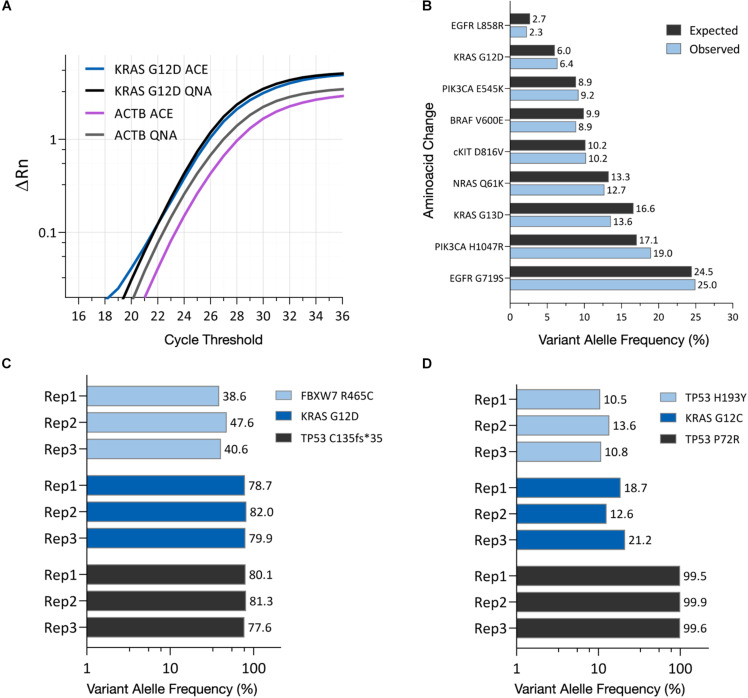
ACE-captured and isolated DNA can be amplified using PCR and NGS. **(A)** qPCR amplification profiles for the *KRAS* G12D mutation and *ACTB* housekeeping gene using DNA isolated from AsPC-1 cell culture EVs using either the ACE chip or the QIAamp Circulating Nucleic Acid Kit (QNA). **(B)** Variant allele frequency detected in the HD701 quantitative multiplex reference DNA standard after ACE isolation, compared to the expected frequency. **(C)** Variant allele frequency shown for *FBXW7*, *KRAS*, *and TP53* mutations in DNA captured from AsPC-1 cell culture EVs. **(D)** Variant allele frequency shown for *TP53* and *KRAS* point mutations in DNA captured from a lung cancer donor plasma sample (Donor 5734). Each replicate indicates an isolation from one chip and the subsequent creation of one NGS library.

The ability to analyze ACE isolated cfDNA by downstream technologies, such as PCR and NGS, was evaluated using cell culture EVs from the AsPC-1 cell line, known to carry the *KRAS* G12D point mutation, as a model system (Bamford et al., 2004; Deer et al., 2010). The cell culture EVs were spiked into K_2_EDTA plasma at a volumetric ratio of 1:10, with DNA isolated using the ACE chip isolation workflow and the QNA workflow. qPCR amplification plots were positive for the presence of the *KRAS* G12D mutation using DNA isolated by both methods. In addition, the housekeeping gene ACTB was amplified from samples isolated using both methods, confirming the feasibility of the ACE chip DNA isolation workflow for PCR analysis (Figure 5A).

To characterize if the ACE chip captures a broad range of DNA sequences, NGS was performed following ACE isolation of the HD701 DNA quantitative reference standard, with variant allele frequency (VAF) values detected that matched the expected VAFs (Figure 5B). NGS was also used to analyze DNA from the AsPC-1 cell culture EVs using the ACE chip. Single nucleotide variants for *KRAS* G12D, *FBXW7* R465C as well as the frameshift *TP53* C135fs^∗^35 were reproducibly detected from the prepared libraries (Figure 5C). These variants are known to be present in this cell line’s genome (Deer et al., 2010) and in the cell culture EVs.

To verify that similar results are observed when using clinical samples, DNA was isolated using the ACE chip from a lung cancer donor that had previously been characterized by solid tissue biopsy for the point mutations *KRAS* G12C and *TP53* H193Y. Following sequencing, both mutations were reproducibly detected using ACE isolated DNA (Figure 5D). Furthermore, the germline mutation *TP53* P72R was also detected on this sample. These results demonstrate that DNA isolated by the ACE chip can be readily used for NGS library preparation and PCR amplification.

#### ACE Chip Platform Isolates EVs Containing Intact RNA

**FIGURE 6:**
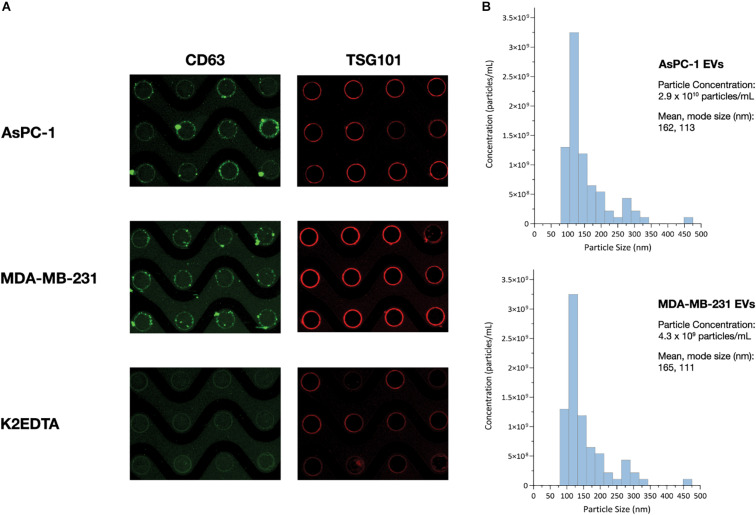
ACE chip enables isolation of EVs and detection of EV-associated protein biomarkers. **(A)** Fluorescence images of the EV transmembrane biomarker CD63 (green), and intra-vesicular biomarker TSG101 (red), following capture of cell culture EVs from the AsPC-1 **(top),** and MB-231 cell lines **(middle)** spiked into K_2_EDTA plasma as well as K_2_EDTA plasma with no EVs spiked **(bottom)**. **(B)** Particle sizing of EVs following ACE capture and isolation using NTA.

The detection of CD63-positive material collected by the ACE chip electrodes (Figure 3) suggested that EVs had been captured on the edge of the electrodes. To further validate the capture of EVs, a luminal EV-associated protein, TSG101, was examined along with the membrane protein CD63, as recommended by ISEV (Théry et al., 2018), using cell culture EVs from MDA-MB-231 and AsPC-1 cell lines. Cell culture EVs were spiked into K_2_EDTA plasma at a volumetric ratio of 1:10 and captured using the detection workflow. Additionally, K_2_EDTA plasma without EV spike was tested using the visualization workflow. Intact membranes block access of antibodies to luminal proteins. To overcome this limitation, EV membranes were permeabilized with a 0.1% saponin solution prior to TSG101 antibody incubation. All three samples demonstrated positive staining for both CD63 and TSG101 (Figure 6A). While the samples with EV spikes demonstrated high florescent intensity for both markers, the non-contrived K2EDTA plasma sample yielded uniformly positive faint signal, likely due to lesser amount of EVs.

**FIGURE 7:**
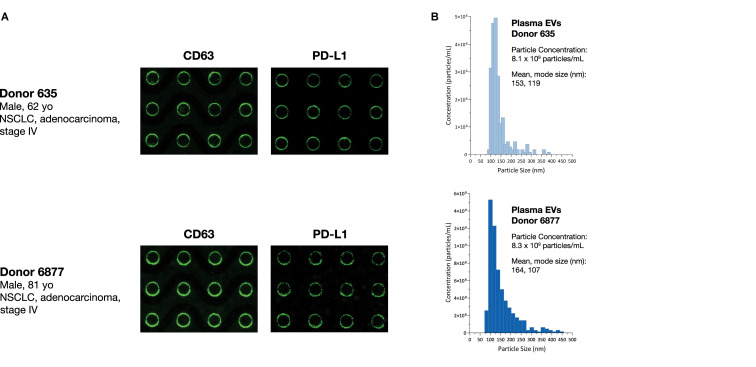
ACE chip enables capture of EVs and detection of EV-associated protein biomarkers from cancer donor plasma samples. **(A)** EVs were captured using the ACE chip from two lung cancer donor samples (Donor 635, top, and Donor 6877, bottom) and antibodies against immune checkpoint protein PD-L1 (**A**, right) were used. EVs were eluted off the chip; EV capture was confirmed by CD-63 staining (**A**, left) and particle sizing **(B)**.

To test the ability of the Verita platform to capture and detect EV-associated cancer biomarkers directly from donor plasma, lung cancer and pancreatic cancer donor plasma was subjected to the ACE detection workflow, and chips were stained with antibodies for Program Death Ligand-1 (PD-L1) or Glypican 1 (GPC-1), respectively. Both sample types were positive for the CD63 EV marker as well as the disease-related EV-associated markers as shown in Figure 7A.

**FIGURE 8:**
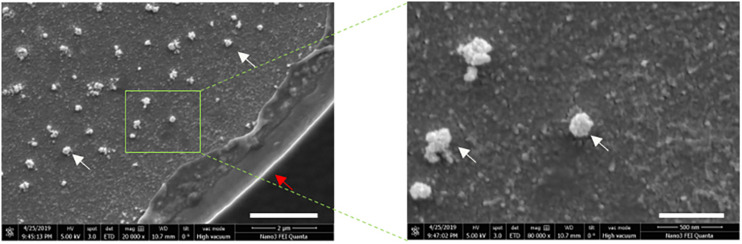
Exosomes remain intact following ACE capture. Scanning electron microscopy (SEM) images of captured cell culture EVS from MDA-MB-231 cells at 20,000× magnification **(left)**, and 80,000× magnification **(right)**. The scale bars correspond to 2 μm and 500 nm, respectively. White arrows point to the EV structures and red arrow to the electrode edge.

To characterize the size distribution of EVs captured by the ACE chip, cell culture EVs from MDA-MB-231 and AsPC-1 cell lines were used as model systems. EVs were spiked at a 1:10 volumetric ratio into K_2_EDTA plasma and purified using the ACE chip isolation workflow, followed by particle size characterization. EVs isolated by the ACE chip exhibited a shift in the distribution of mean particle size compared to pre-isolated material for MDA-MB-231 (136–165 nm), while there was no significant shift for the AsPC-1 EVs, as shown in Figure 6B and Supplementary Figure S3. To test the ability of the ACE chip to isolate EVs from donor samples, particle size characterization was performed with eluates from lung cancer and melanoma donor plasma samples (Figure 7B). Mean particle sizes were 164 and 147 nm, respectively, which reflected the size of EVs purified from cell lines (range 136–165 nm) and implied the isolation of intact EVs using the ACE chip. The EVs captured from the MDA-MB-231 cell line were also examined with scanning electron microscopy (SEM) as shown in Figure 8. Vesicles with a spherical and/or peaked-spherical morphology, as previously noted (Ibsen et al., 2017; Lewis et al., 2020), were observed to decorate both the top and bottom of the electrode edges. The structures observed are in the range of 100–200 nm in concordance with the particle sizing measurements while the less-than perfect spherical form may be attributed to protein aggregation around the vesicles.

**FIGURE 9:**
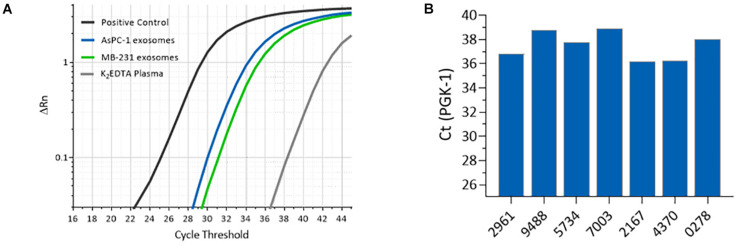
Amplification of mRNA isolated from ACE-captured EVs. **(A)** RT-PCR amplification profiles for housekeeping mRNA gene *PGK1* using ACE isolated materials from K_2_EDTA plasma spiked with MDA-MB-231 cell culture EVs (green), AsPC-1 cell culture EVs (blue), and with no spike (gray). **(B)** RT-PCR for *PGK1* from endogenous EVs captured from melanoma and lung cancer donor K_2_EDTA plasma samples.

EVs have been reported to contain diverse types of RNA, and to protect the RNA from degradation by RNases (Rak and Guha, 2012; Taylor and Gercel-Taylor, 2013; Enderle et al., 2015; Zhang et al., 2015). Plasma samples were processed using the ACE chip to isolate EVs, which were tested for the presence of RNA using RT-PCR for the housekeeping mRNA gene *PGK1*. Initially, AsPC-1 and MDA-MB-231 cell culture EVs were spiked at a 1:10 volumetric ratio into K_2_EDTA plasma as well as K_2_EDTA plasma without any spiked material. Isolates from both spike and no spike experiments displayed strong signals in RT-PCR (Ct = 30 and Ct = 31, for AsPC-1 and MDA-MB-231 derived EVs, respectively), with no spike control demonstrating a weaker positive signal (Ct = 38.3), which likely represents amplification of RNA from endogenous EVs (Figure 9A). Additionally, EVs isolated from six lung cancer donor samples and one melanoma donor sample were tested by RT-PCR, and all showed positive amplification, whereas the no-template control showed no amplification up to 45 cycles (Figure 9B). Amplification performed with *PGK1* exon-spanning primer pairs failed to generate a product from gDNA, confirming that the signal observed was not generated by contaminating gDNA (data not shown). These data demonstrated that the ACE chip captured EVs containing intact RNA can be probed using RT-PCR.

## Discussion

The escalating adoption of a precision medicine approach, particularly for oncology applications, continues to push the demand for improved isolation and detection workflows, both for clinical and research discovery applications. This study focused on evaluating applicability of the platform as an on-chip detection tool and/or as a flexible isolation workflow to analyze the three classes of biomarkers that are critical for precision medicine: cell-free DNA, RNA, and exosomal proteins.

Cell-free DNA has gained recognition as an important cancer molecular biomarker and its concentration levels appear to reflect apoptotic and necrotic cell death as well as inflammatory and vascularization processes within the tumor and the surrounding tissues. Previous studies have demonstrated that elevated cfDNA levels within the blood occur for cancers originating from different tissues, including lung (Ulivi et al., 2013; Bortolin et al., 2015; Szpechcinski et al., 2015), ovary (Kamat et al., 2006; No et al., 2012), breast (Umetani et al., 2006; Lehner et al., 2013; Cheng et al., 2017), and others (Bettegowda et al., 2014; Mouliere et al., 2014). A fraction of the cfDNA released into the blood stream is a product of non-programmed cell death, e.g., necrosis, resulting in long circulating cfDNA fragments that have been suggested to be elevated in solid tumors (Marzese et al., 2013). The levels of long circulating cfDNA can be used to monitor treatment response, tumor progression, or discriminate healthy controls from benign or cancerous conditions (Umetani et al., 2006; Agostini et al., 2011; Pinzani et al., 2011; Zonta et al., 2015; Szpechcinski et al., 2016). In this work, we demonstrated how the ACE-based method preferentially selects and quantifies a fraction of DNA that is greater than 300 bp. This offers potential to evaluate the impact of biological processes on a fraction of cfDNA that is typically not associated with apoptotic origin.

The ACE chip also produces purified DNA that is compatible with downstream molecular analysis techniques such as PCR and NGS. The DNA isolated from a quantitative reference standard, HD701, produced VAF values in the expected range, and cell culture EVs from the AsPC-1 cell line also displayed known genomic variants. According to the literature, the *FBXW7* R564C variant is heterozygous while the *KRAS* G12D and *TP53* c135fs^∗^35 variants are homozygous. While the VAF values observed here were not exactly 50 and 100%, respectively, there is a consistent trend in zygosity (∼40% VAF for *FBXW7* and ∼80% for *KRAS* and *TP53*). Other analysis techniques, such as digital droplet PCR, may help validate this observation. Additionally, variant alleles that were initially identified in a lung cancer tumor tissue biopsy were selectively amplified from cfDNA isolated by ACE from the corresponding plasma sample. Several studies have previously suggested the possibility of detection, treatment monitoring, and prognostics using cfDNA (Dawson et al., 2013; Bronkhorst et al., 2019). The Verita platform may support these efforts through both the measurement and isolation of cfDNA. The significance of the selectivity toward long base-pair cfDNA by the ACE chip is currently under investigation, and further studies may elucidate the clinical relevance of this fraction of the cfDNA population, as has been previously suggested by DNA integrity index studies (Cheng et al., 2009; Catarino et al., 2012; Madhavan et al., 2014).

EV studies represent a growing field of translational research and development because of their capacity to act as biomarkers for cancer progression, angiogenesis, immune suppression, and potential therapeutic uses (Lo Cicero et al., 2015; Reclusa et al., 2017; Castellanos-Rizaldos et al., 2019). Common EV markers (CD63, TSG101) and disease-specific EV-associated proteins (PD-L1, GPC-1) were readily detected following isolation by the ACE chip from either spiked EV or donor cancer samples. The detection workflow described here employed two simultaneous markers imaged at two different fluorescent wavelengths, directly from biological fluids, without sample preprocessing.

The isolation of EVs on the ACE chip was confirmed by both SEM and particle size analyses following isolation. The former method revealed semispherical bodies on the edge of the electrodes with sizes from 100 to 200 nm while the latter method showed the expected size distribution for EVs. Nevertheless, the true size distribution of EVs in particular exosomes is still in debate and is heavily influenced by the isolation method used (ultracentrifugation, precipitation, and affinity capture) (Cvjetkovic et al., 2014; Van Deun et al., 2014). The particle size distribution for the MDA-MB-231 EVs shown here is similar to that shown by other particle sizing studies (Jenjaroenpun et al., 2013; Kowal et al., 2016), and it is minimally altered following capture and isolation with the ACE chip, suggesting that ACE does not disturb the physicochemical characteristics of the EVs. When endogenous EVs from cancer donor samples were isolated with the ACE chip, similar particle size distributions were observed, confirming that the properties observed for the control models can be extended to donor samples. Although most of the size distribution is centered around 120 nm, there are a few “peaks” at larger particle sizes >300 nm (Figure 8) that may arise from microvesicles isolated on the ACE chip and/or due to the fact that there is no plasma prefiltering prior to running the isolation workflow, in contrast to other EV extraction methodologies (Enderle et al., 2015).

RT-PCR of *PGK1* mRNAs purified by ACE from both spiked and endogenous plasma EVs generated the expected PCR products, suggesting that the quality of the ACE chip-isolated material is sufficient for downstream transcriptomics. There is a distinct difference between the amplification values from the spiked EVs compared to the K_2_EDTA plasma without a spike. The amplification observed for the non-spiked plasma is likely due to the presence of endogenous EVs that are also isolated on the ACE chip. Likewise, EVs isolated from cancer plasma donors show positive amplification on RT-PCR. Interestingly, the RT-PCR amplification was performed without prior RNA extraction from the ACE chip-isolated EVs. It is likely that the ACE EV isolation method reduced the level of contaminating proteins that may interfere with the amplification process, as observed from the Protein Bioanalyzer results (Supplementary Figure S4). This may allow the reverse transcription process more access to the RNA cargo compared to traditional EV isolation methods. In the future, the ACE-based chip EV isolation method may facilitate the use of EV-derived RNA to determine tissue of origin and other clinical applications (Kalluri and LeBleu, 2020).

While this work was largely focused on cancer, it is important to note that the ACE-based biomarker isolation platform is disease-agnostic. The platform demonstrated its potential to expand the discovery toolset—with the ability to simultaneously investigate multiple classes of unprocessed biomarkers in the same sample—potentially offering a more comprehensive understanding of disease biology for the next generation of personalized medicine spanning many therapeutic applications beyond oncology.

## Data Availability Statement

All data requests will promptly undergo an internal review to verify whether the request is subject to any intellectual property or confidentiality obligations. Any released data and materials will be subject to a data transfer agreement. Requests to access the datasets should be directed to the corresponding authors.

## Ethics Statement

The studies involving human participants were reviewed and approved by the Western IRB. The patients/participants provided their written informed consent to participate in this study.

## Author Contributions

JH, DB, and RK designed the experiments. DS, JL, AK, OP, KT, and DB performed the experiments. JH, JL, ID, HB, and DH analyzed the results and provided insights. JH, JL, RT, HB, IC, DH, and RK wrote the manuscript. All authors contributed to the study design, advised on data analysis, provided insights, and provided final approval of the manuscript.

## Conflict of Interest

JH, DS, JL, AK, OP, ID, RT, HB, IC, and RK were full-time employees of Biological Dynamics. DH was a member of the Biological Dynamics Scientific Advisory Board. The authors declare that this study received funding from Biological Dynamics. The funder had the following involvement in the study: study design, collection, analysis, interpretation of data, the writing of this article and the decision to submit it for publication. The remaining authors declare that the research was conducted in the absence of any commercial or financial relationships that could be construed as a potential conflict of interest.
