# Supplementary material for: Simultaneous Isolation of Circulating Nucleic Acids and EV-Associated Protein Biomarkers From Unprocessed Plasma Using an AC Electrokinetics-Based Platform
Source: Front Bioeng Biotechnol. 2020 Nov 5;8:581157. doi: 10.3389/fbioe.2020.581157 (PMC7674311; doi:10.3389/fbioe.2020.581157)
Supplement: Supplementary file 1 [file Data_Sheet_1.docx]

**SUPPLEMENTAL INFORMATION**

TITLE

Simultaneous Isolation of circulating nucleic acids and EV-associated protein biomarkers from unprocessed plasma using an AC Electrokinetics-based platform

AUTHORS

Juan Pablo Hinestrosa^1^ (J.P.H.), David J. Searson^1^ (D.J.S.), Jean M. Lewis^1^ (J.M.L.), Alfred Kinana^1^ (A.K.), Orlando Perrera^1^ (O.P.), Irina Dobrovolskaia (I.D.), Kevin Tran^2^ (K.T.), Robert Turner^1^ (R.T.), Heath I. Balcer^1^ (H.I.B.), Iryna Clark^1^ (I.C.), David Bodkin^3^ (D.B.), Dave S. B. Hoon^2^ (D.S.B.H.), and Rajaram Krishnan^1*^ (R.K.)

AFFILIATION

^1^Biological Dynamics, Inc., San Diego, CA

^2^Departments of Translational Molecular Medicine and Sequence Center, John Wayne Cancer Institute, Santa Monica, CA

^3^Cancer Center Oncology Medical Group, La Mesa, CA

CORRESPONDING AUTHOR

*Rajaram Krishnan, PhD

raj@biologicaldynamics.com

**AC Electrokinetics (ACE) Principles**

AC Electrokinetics (ACE) is the combination of three phenomena: dielectrophoresis (DEP), AC Electrothermal flow (ACET) and AC electroosmotic flow (ACEO). Given that the media used on the ACE chip is relatively of high conductivity (~ 10mS/cm), the contribution from ACEO is negligible(Green and Nili, 2012). By applying a set of voltages (*V*) and frequencies (*ω*) (as described in the main manuscript, materials and methods section), nanoparticles from 10 to 1000 nm can be isolated on a platinum microelectrode array while larger particles, i.e., cells, are moved away from the electric field region. Small molecules, such as serum albumin, are unaffected by the electric field. An illustration of the DEP and ACET phenomena, the electrode rings and the interdigitated array are shown in Figure S1.


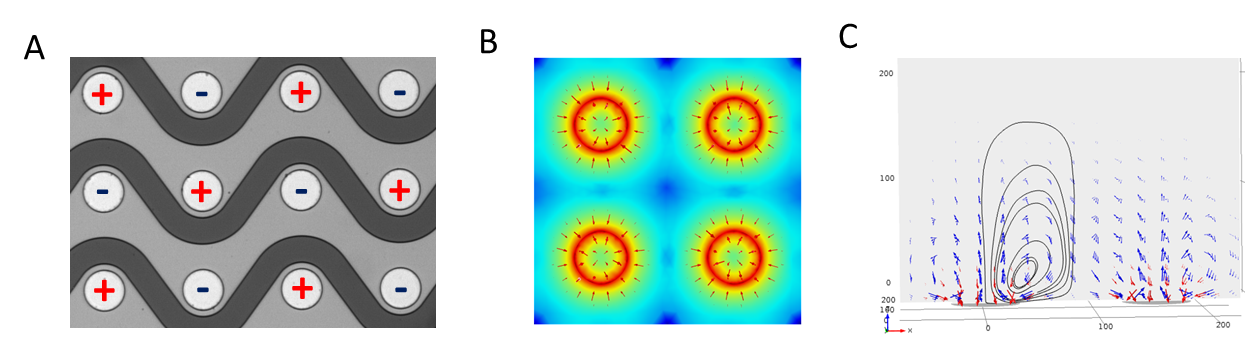


**Figure S1.** **AC Electrokinetics.** (A) Depiction of the interdigitated array showing relative polarity, each electrode is opposite to that of its nearest neighbor. (B) Schematic of concentration of *F_DEP_* on the edge of the electrodes giving rise to the capture patter observed for cfDNA and EVs. (C) Schematic of the *F_DEP_* (red arrows) and the *F_ACET_* (, blue arrows and black lines) on the microelectrode array and the sample media.

The Dielectrophoretic force (*F_DEP_*) is concentrated on the edges of the electrode, it is of short range (~ 5 µm in the Z-direction) and it is proportional to the ~ *V*^2^. The AC electrothermal force (*F_ACET_*) is driven by a thermal gradient that arises within the fluid as an external electric field is applied. The ACET extends in the Z-direction ~ 60 µm and ~ V4. The ACET is responsible for mixing inside the chamber, while the DEP force allows for capture of nanoparticles.

For a spherical particle of radius *r*, the time-averaged *F_DEP_* equation is(Oh et al., 2009; Lu et al., 2015):

$F_{DEP}=2\pi\varepsilon_{m}r^{3}Re\left[ K\left( \omega\right) \right]\nabla E_{RMS}^{2}$ (1)

Where *ε_m_* is the permittivity of the media, $\nabla E_{RMS}^{2}$ is the gradient of the root-mean-squared electric field (E_RMS_), and the real part of the Clausius-Mossoti factor, *K(ω)*, is defined as:(Lu et al. 2015)

$Re\left[ K\left( \omega\right) \right]=Re\left( \frac{\varepsilon_{p}^{*}-\varepsilon_{m}^{*}}{\varepsilon_{p}^{*}+2\varepsilon_{m}^{*}} \right)$ (2)

where $\varepsilon_{p}^{*}$ and $\varepsilon_{m}^{*}$ are the complex permittivities of the particle and media respectively, defined by:

$\varepsilon^{*}=\varepsilon-\frac{j\sigma}{\omega}$ (3)

here *j*^2^ = -1, *ε* is the dielectric constant and σ is the conductivity. If the particle has a positive *K(ω)*, it will experience positive *F_DEP_* and move toward the electrodes.

The ACET is driven by the passage of current thru the media, causing Joule heating according to the following equation(Oh et al., 2009):

$F_{ACET}=\frac{\varepsilon_{m}}{2}\left[ -2.4\%C^{-1}\frac{\nabla T.E}{1+\left( \omega\tau\right)^{2}}E+0.2\%C^{-1}\left| E^{2} \right|\nabla\varepsilon_{m} \right]$ (4)

Where T is the media temperature and t is the charge relaxation time between the media permittivity (ε_m_) and the media conductivity (σ_m_). A conductivity increase leads to greater F_ACET_ and that effects is exploided in the ACE chip for mixing of particles in the fluid chamber.

The ACE chip used on the Verita platform is a micro-electromechanical systems (MEMS) fabricated device with dimensions of 14 mm x 52 mm organized into an 8-array configuration, with each array consisting of ~ 1000 platinum circular electrodes (for visualization workflow) or a single 1-chamber array for the isolation workflow. In all cases, the ACE chip coated with a hydrogel as seen in Figure 2 of the main manuscript and described in previous publications(Turner et al., 2018).

**Table S1. Clinical characteristics of cancer donors**

| **sample ID** | **Matrix** | **Cancer Type** | **Histology** | **Stage** | **Age** | **Gender** | **Sample Source** |
| --- | --- | --- | --- | --- | --- | --- | --- |
| 635 | K_2_EDTA | NSCLC | Adenocarcinoma | IV | 62 | Male | IRB (internal collection) |
| 6877 | K_2_EDTA | NSCLC | Adenocarcinoma | IV | 81 | Male | IRB (internal collection) |
| 5734 | K_2_EDTA | NSCLC | Squamous Cell Carcinoma | IV | 56 | Male | IRB (internal collection) |
| 2961 | K_2_EDTA | NSCLC | Adenocarcinoma | IV | 62 | Female | IRB (internal collection) |
| 4160 | K_2_EDTA | NSCLC | Adenocarcinoma | III | 78 | Male | IRB (internal collection) |
| 9488 | K_2_EDTA | NSCLC | Adenocarcinoma | IV | 80 | Male | IRB (internal collection) |
| 0117 | K_2_EDTA | Pancreatic | N/A | IIA | 73 | Male | DLS (biobank) |
| 7003 | K_2_EDTA | Melanoma | N/A | IIC | 68 | Female | BioIVT (biobank) |
| 4370 | K_2_EDTA | NSCLC | Adenocarcinoma | IV | 62 | Female | IRB (internal collection) |
| 2167 | K_2_EDTA | NSCLC | N/A | IV | 62 | Female | IRB (internal collection) |
| 0278 | K_2_EDTA | NSCLC | Squamous Cell Carcinoma | IIA | 68 | Male | BioIVT (biobank) |

**DNA Isolation from Different Matrices**

**
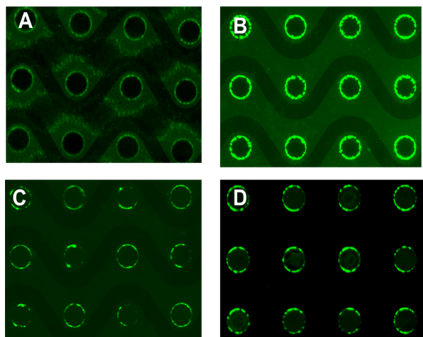
**

**Figure S2.** Fluorescence imaging/Visualization of gDNA stained with YOYO-1 dye from commonly collected human biofluids. gDNA (50 pg/µL) and YOYO-1 (1:5000 dilution) were spiked into (A) whole blood, (B) serum, (C) plasma, and (D) cerebrospinal fluid followed by capture on the ACE chip.

**EV Particle Sizing Before ACE**

**
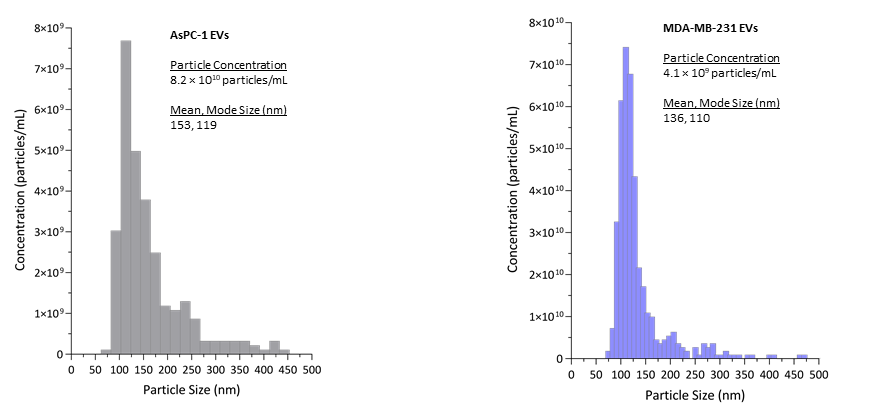
**

**Figure S3.** Particle sizing of EVs prior to ACE capture and isolation using NTA for AsPC-1 (left) and MDA-MB-231 (right) EVs.

**Protein Contamination Analysis**

**
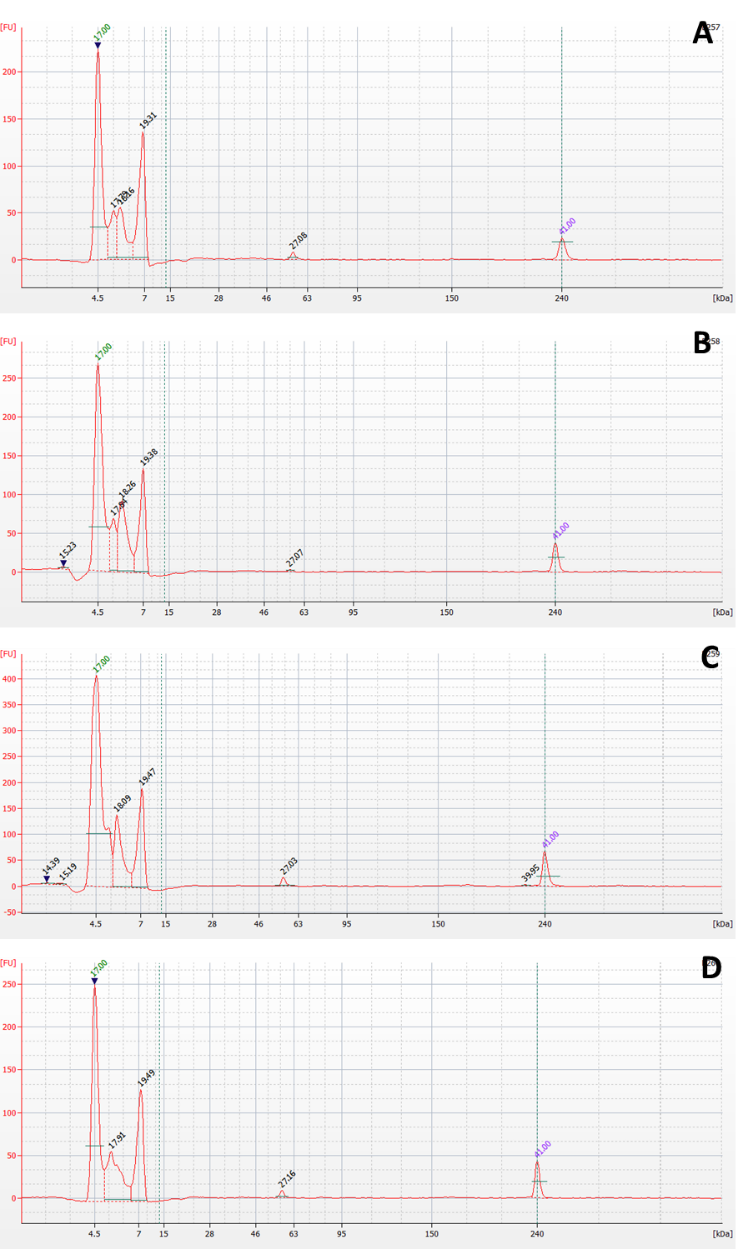
**

**Figure S4.** Protein 2100 bioanalyzer trace for ACE isolated material from the K_2_EDTA plasma of a non-small cell lung cancer (Donor 5734). The small peak observed at ~57 kDa is from human serum albumin, and the calculated concentration is 18.6 ng/μL.

**References**

Green, N.G., and Nili, H. (2012). "Dielectrophoresis," in *Encyclopedia of Nanotechnology,* ed. B. Bhushan. (Dordrecht: Springer Netherlands), 534-543.

Lu, Y., Liu, T., Lamanda, A.C., Sin, M.L., Gau, V., Liao, J.C., et al. (2015). AC Electrokinetics of Physiological Fluids for Biomedical Applications. *J Lab Autom* 20(6)**,** 611-620. doi: 10.1177/2211068214560904.

Oh, J., Hart, R., Capurro, J., and Noh, H.M. (2009). Comprehensive analysis of particle motion under non-uniform AC electric fields in a microchannel. *Lab Chip* 9(1)**,** 62-78. doi: 10.1039/b801594e.

Turner, R., Madsen, J., Herrera, P.D.S., Wallace, J., Madrigal, J., Hinestrosa, J.P., et al. (2018). Cancer Detection at your Fingertips: Smartphone-Enabled DNA Testing. *2018 40th Annual International Conference of the IEEE Engineering in Medicine and Biology Society (EMBC)***,** 5418-5421.
